# A Systematic Review of EEG and MRI Features for Predicting Long-Term Neurological Outcomes in Cooled Neonates With Hypoxic-Ischemic Encephalopathy (HIE)

**DOI:** 10.7759/cureus.71431

**Published:** 2024-10-14

**Authors:** Sylvia Edoigiawerie, Julia Henry, Naoum Issa, Henry David

**Affiliations:** 1 Neurology, University of Chicago Medical Center, Chicago, USA; 2 Pediatric Neurology, AdventHealth Medical Group, Orlando, USA; 3 Neurological Surgery, University of Chicago Medical Center, Chicago, USA; 4 Pediatric Neurology, University of Chicago Medical Center, Chicago, USA

**Keywords:** brain cooling, cooling therapy for asphyxiated neonates, eeg in children, electroencephalography (eeg), epilepsy research, high risk neonates, hypoxic brain injury, hypoxic-ischemic encephalopathy, neonatal stroke, peripartum prognostication

## Abstract

Neonatal hypoxic-ischemic encephalopathy (HIE) represents a significant global disease burden, but more importantly, it leaves a lasting impact of disability on individual children and their families. HIE outcome prognostication is important for guiding clinical interventions and counseling families. The objective of this study was to systematically review early electroencephalogram (EEG) and magnetic resonance imaging (MRI) features associated with long-term neurological outcomes in infants after perinatal HIE. Articles were extracted from PubMed, CINAHL, and Scopus. Twenty studies were included that assessed EEG and/or MRI patterns in neonates who underwent therapeutic hypothermia and were followed to determine long-term outcomes. Articles that did not meet the inclusion criteria were excluded. Covidence review manager (Melbourne, Australia: Covidence) was used to extract, evaluate, and synthesize review results. Of the articles included, eight focused on EEG features, eight on MRI features, and four on assessments using both EEG and MRI. Abnormal EEG background and burst suppression severity were associated with poor outcomes. Higher MRI injury scores in the basal ganglia and thalamus were also correlated with poor outcomes. Finally, studies also revealed restricted diffusion and greater lesion size in the subcortical gray matter correlated with poor outcomes. We also identified limitations in the included studies which primarily involved sample size, potential for MRI pseudonormalization, and the potential tradeoff between retention of infants able to receive long-term follow-up and attrition of those lost to follow-up.

We conclude that EEG background patterns, MRI scoring, subcortical lesion burden, and MRI diffusivity are sensitive metrics for predicting outcomes. Both early EEG and MRI features may serve as high-fidelity biomarkers for secondary energy failure and for counseling families of neonates at high risk for devastating neurologic outcomes. Additionally, there is a paucity of information on the impact of HIE on brain areas outside of the standard clinical basal-ganglia and watershed patterns, especially in locations like the corpus callosum. Finally, MRI pseudonormalization may underestimate the extent of injury in these studies.

## Introduction and background

Neonatal hypoxic-ischemic encephalopathy (HIE) results from prolonged perinatal hypoxia related to intrauterine factors, delivery complications, or the need for significant resuscitation at birth. It represents a significant global disease burden [1,2]. With an estimated incidence of 1.5 per 1000 births, it accounts for nearly a quarter of infant deaths worldwide [3,4]. In addition to mortality, nearly 60% of HIE cases result in severe disability such as cerebral palsy [1,2]. Birth asphyxia is among the top three global causes of disability-adjusted life years (DALYs), and in 2010 caused 42 million DALYs, twice the estimated disability burden of diabetes [5,6].

HIE pathophysiology can be divided into four stages. In the first stage, primary energy failure, anaerobic metabolism predominates in the brain due to lack of oxygen, and if prolonged, it progresses to excitotoxicity and neuronal death through the failure of Na^+^/K^+^ pumps [7,8]. After primary energy failure, there is a latent phase, a variable-duration period during which therapeutic hypothermia is used in an attempt to reduce the magnitude of neuronal loss [8]. During the latent phase, there is a transient return of normal cerebral perfusion and partial recovery of neuronal damage [9]. Secondary energy failure then ensues and is characterized by delayed neuronal cell death, cytotoxic edema, excitotoxicity, and microglial activation, occurring 6-48 hours after injury [7,8]. Decreased cellular metabolism and widespread neuronal cell death during secondary energy failure may manifest on the EEG as burst suppression and/or reduced amplitudes [10]. The majority of neonatal seizures also start during this stage [11]. In a subset of patients, tertiary brain injury follows secondary energy failure, and results in further reduction of neuronal cell counts and astrogliosis [7,8].

HIE outcome prognostication is important for guiding clinical interventions and counseling families. Here, 21 articles that evaluate prognostic features from magnetic resonance imaging (MRI) within the first few weeks of life and EEG during and/or shortly after therapeutic hypothermia are reviewed. To the best of our knowledge, there hasn't been a prior study that attempts to correlate specific MRI injury patterns with EEG patterns in HIE status post-brain cooling. This study aimed to provide the bedside clinician with higher-quality prognostication by systematically characterizing the sensitive and specific features and patterns from routine EEG and MRI data.

## Review

Methods

Search Strategy

**Figure 1:**
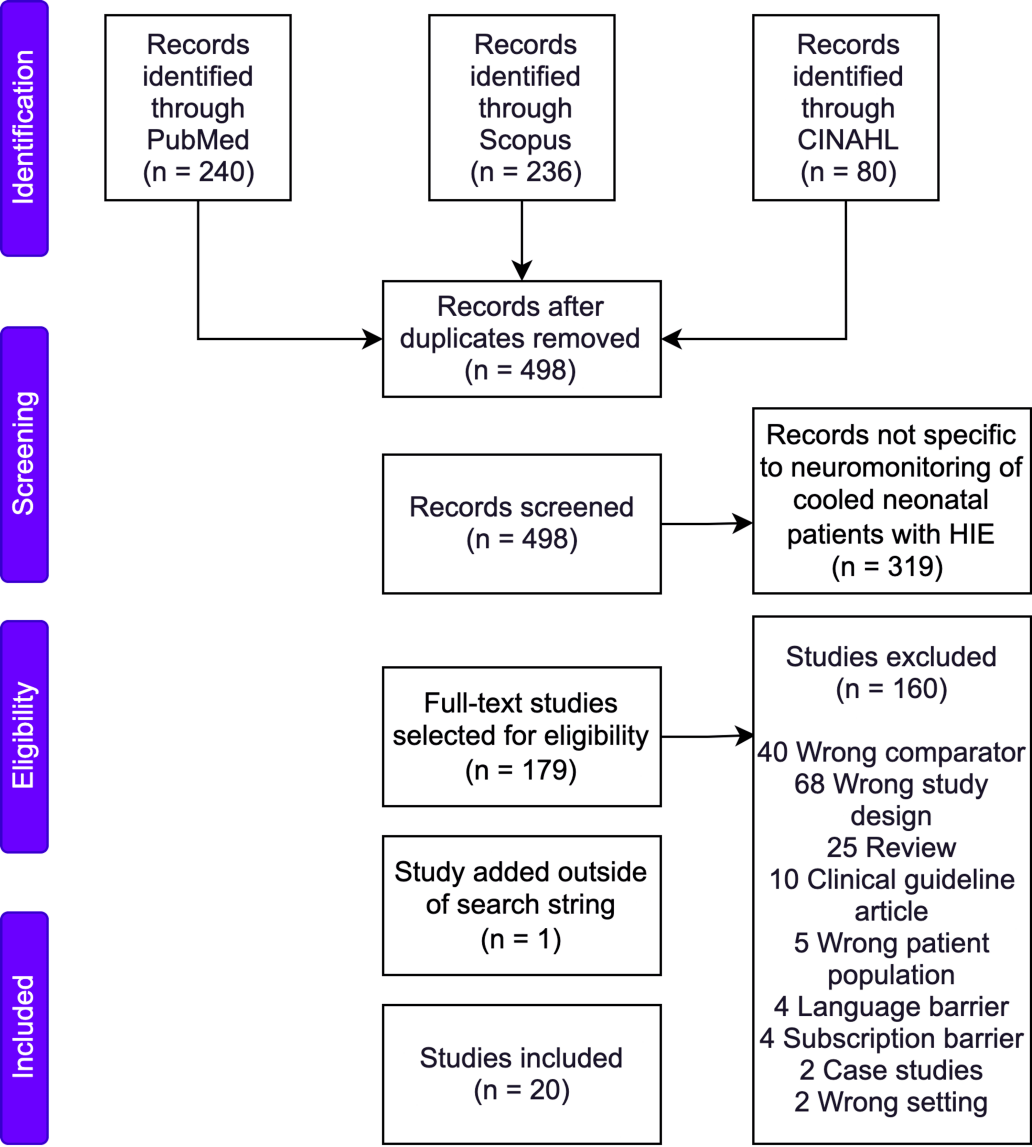
PRISMA flow diagram depicting article selection criteria. PRISMA: Preferred Reporting Items for Systematic Reviews and Meta-Analysis

Articles were collected from the following three databases: PubMed, CINAHL, and Scopus using the Preferred Reporting Items for Systematic Reviews and Meta-Analysis (PRISMA) principles (Figure 1) (appendix 1 and 2) [12]. This search was formulated using the Population, Intervention, Comparison, and Outcome (PICO) framework [13]. A reproducible search string is shown in appendix 3. Titles were screened by authors SE and HD using the data extraction and screening tool Covidence (Melbourne, Australia: Covidence) [14].

Inclusion and Exclusion Criteria

Articles were included if they reported a median follow-up period of ≥18 months for neurodevelopmental outcomes in patients who received therapeutic hypothermia and underwent EEG monitoring during the therapy, or if they conducted MRI scans within the first month of life. Assessments of functional outcomes are typically performed after 18 months of age in neonatal brain injury.

Studies were excluded if they did not report established metrics for functional outcomes, such as Bayley Scales of Infant Development (BSID), Griffiths Mental Development Scales, Gross Motor Function Classification System, the development of cerebral palsy, or post-neonatal epilepsy rate [15-17]. Studies with only nonfunctional outcomes, such as length of hospital stay or post-neonatal MRI results, were excluded. One study, Trivedi et al., was identified from another study, Rusli et al., which was captured by the search criteria [18,19].

Results

Of the 498 studies initially screened, 20 met the inclusion criteria (Figure 1). Sixteen studies were retrospective, three were prospective, and one was a multicenter randomized control trial (appendix 4-6). One prospective study, Trivedi et al., was identified from other included articles, independent of the search criteria [18]. The included articles are presented in three following themes: (1) electrographic features associated with HIE outcomes, (2) MRI features associated with HIE outcomes, and (3) a combination of MRI and electrographic features associated with HIE outcomes. Most articles (n=15) assessed outcomes using either the BSID-II or BSID-III scores. The BSID measures cognitive development with composite scores below 70 in the BSID-II and below 80 in the BSID-III signifying a risk of poor neurodevelopmental outcome [20]. Eight of the 12 MRI-related articles conducted MRI scans within the first 10 days of life (appendix 5 and 6).

A summary of each article’s study design, setting, patients included, outcome criteria, and features assessed is included in appendix 4-6. EEG and MRI features found to be consistently associated with poor long-term outcomes are summarized in Table 1.

**Table 1:** Summary of features associated with abnormal outcome in infants that have undergone therapeutic hypothermia. LV: low voltage; FT: flat trace; DNV: discontinuous normal voltage; CNV: continuous normal voltage; ADC: apparent diffusion coefficient; MRI: magnetic resonance imaging; NICHD: National Institute of Child Health and Human Development

Modality	Features significantly associated with abnormal outcome
EEG	EEG and aEEG background pattern (LV, FT, no DNV, no CNV) [21-24], interburst intervals >30s [23], low interburst interval amplitudes (<15 μV), high cortical burst power post-therapeutic hypothermia day three [22], lack of sleep-wake cycles during hypothermia [25], total seizure burden during hypothermia [11,21,24,26,27]
MRI	MRI injury pattern/severity score (using NICHD scoring system) [27,28], MRI lesion volume [28], restricted diffusion in the basal ganglia, thalamus, and splenium of the corpus callosum [29,30], lack of week two MRI pseudo normalization [31]

EEG Features Associated With HIE Outcomes

EEG qualitative assessments: Two studies, Weeke et al. and Fitzgerald et al., evaluated EEG background qualitatively based on visual assessment during hypothermia [21,24]. They graded the EEG background using ordinal scales. The lowest grade was given to EEGs with mild discontinuity (interburst interval duration ≤10 seconds) for gestational age, while the highest grade was given to very abnormal EEG backgrounds with severe discontinuities (interburst interval duration ≥30 seconds) and attenuated voltage (<25 μV). Both articles found that a severely abnormal EEG background within the first 24 and 36 hours of life was associated with abnormal outcomes [21,24].

Burst and interburst patterns: Koskela et al. 2021 assessed EEG bursts quantitatively [22]. They computed the burst power between 8 and 30 Hz in the hours directly following therapeutic hypothermia (≥post-natal day three). They found that elevated bilateral central, occipital, and right temporal burst power (channels C3, C4, O1, O2, and T4) was inversely correlated with BSID-III language and motor scores with correlation coefficients (R-values) ranging from -0.49 to -0.31. High burst power directly after hypothermia was therefore associated with worse BSID-III scores [22].

Dereymaeker et al. used an automated assessment of interburst intervals (IBIs) to grade EEGs during therapeutic hypothermia in 19 neonates [23]. These bursts, by definition, are abnormal and not the pattern seen in tracé alternant [32]. They used a metric called dynamic IBIs (dIBIs), which measures both IBI duration and amplitude, to score the severity of EEG abnormality from one to five. EEGs with high-amplitude and short-duration IBIs were assigned a low severity score while EEGs with low-amplitude and long-duration IBIs were assigned a high severity score. They found that at 19-24 hours of life, median IBI duration <10 seconds, and IBI amplitudes ≥15 µV were associated with favorable outcomes (p<0.001).

Amplitude-integrated EEG (aEEG): Five articles evaluated aEEG background during at least one hour of therapeutic hypothermia for outcome prognostication [26,27,33-35]. All five articles used the BSID-II or BSID-III at 18-24 months as outcome metrics. Four studies found that any of the most abnormal aEEG patterns, including burst-suppression (BS), low voltage (LV), or a flat trace (FT), were associated with poor outcomes (p<0.05) [26,27,33,35]. In addition, Csekő et al. reported that an abnormal aEEG at 60 hours of life had a positive predictive value of 0.92 for poor outcomes [33].

Interhemispheric dynamics: Leroy-Terquem et al. 2017 evaluated EEG asynchrony as a predictor of outcomes using the World Health Organization (WHO) disability score in 40-term neonates [36]. Asynchrony was defined as a discontinuous background with periods of abnormal burst activity with burst onsets separated by at least 1.5 seconds over both hemispheres [36]. EEG discontinuities identified were noted to be pathologic and independent of the normal tracé alternant pattern observed in the quiet sleep of term neonates [32]. Asynchrony was assessed within the first 48 hours of life and again from another day within the first week of life. Asynchrony during the first seven days of life identified neonates who would develop major disabilities with a sensitivity of 80% and a specificity of 97%.

Sleep-wake cycles: Takenouchi et al. evaluated EEGs following therapeutic hypothermia from 72 to 144 hours of life for evidence of sleep-wake cycles (SWCs) in 29 neonates [25]. Neurocognitive outcomes were based on the BSID-II Mental Development Index (MDI). Takenouchi et al. classified a neonate as having SWCs if their EEG contained at least two state changes across a six-hour EEG epoch. These state changes indicated a transition into quiet sleep or wakefulness. Failure to acquire SWCs within the first 120 hours of life had a sensitivity of 90% and a specificity of 60% for poor outcomes (p=0.02).

Seizures: Five studies assessed seizures as predictors of functional outcome [11,21,24,26,27]. Seizure burden was quantified in four of these articles [11,21,26,27]. Basti et al. 2020 found that increasing seizure burden, as assessed on EEG across 30 neonates, was significantly associated with poor outcomes (p=0.0004) [26]. Fitzgerald et al. used a different method for calculating seizure burden, epileptic seizure exposure, which is defined as the total number of seizures in the EEG during both hypothermia therapy and rewarming [24]. The total duration of EEG varied depending on the clinical needs of the child, so seizure rates were not reported. They found that high epileptic seizure exposure (≥4 seizures during cooling and rewarming) was associated with motor delay (p<0.01) and having ≥3 seizures during cooling and rewarming was associated with language delay (p=0.01) using the BSID-III. These studies are consistent in showing that a high seizure burden (>30 min/h or ≥3 seizures total) during therapeutic hypothermia is associated with worse outcomes.

MRI Injury Patterns Associated With HIE Outcomes

Qualitative MRI injury scoring: Eight articles evaluated MRI features alone for predicting outcomes [18,19,28-31,37,38] (appendix 5). Five studies assessed MRI using published injury severity scoring systems [18,19,27,28,38]. One study applied the National Institute of Child Health and Human Development (NICHD) scoring system, which ranks lesion severity across the basal ganglia, thalamus, internal capsule, watershed regions, and cerebral hemispheres [39]. Injury scores span the following six categories: 0, 1A, 1B, 2A, 2B, and 3; zero signifying a normal MRI and three signifying hemispheric devastation. Chang et al. 2020 found that the NICHD scoring system had an area under the curve (AUC) of 0.756 for predicting poor outcomes at 18-24 months. Prognostication using the scoring system was marginally better than simply using lesion size or lesion count, which had AUCs of 0.718 and 0.705, respectively [28]. Lin et al. found that specific patterns of injury were predictive of poor outcomes. MRI lesions scored as 2A and 2B - affecting the basal ganglia/thalamus and the posterior limb of the internal capsule (PLIC) - were linked to poor outcomes (p<0.001). In contrast, lesions without these characteristics were not associated with poor outcomes [27].

In contrast, Mastrangelo et al. used the Bednarek Severity scoring system to characterize the MRI [40]. Like the NICHD score, the Bednarek score measures HIE injury severity, but unlike the NICHD, it sums the individual injury scores across the basal ganglia, brainstem, white matter, cortex, and cerebellum into a single global injury severity score [40]. Mastrangelo et al. suggested that a global MRI injury score of 55 can be used as a cutoff to separate good from poor outcome groups. Bednarek scores below 55 (range: 48-55) were associated with better neuromotor outcomes scores at 24 months, while Bednarek scores above 55 (range: 56-186) were associated with worse neuromotor scores (p=0.02) [38].

One study found that a single MRI-based scoring system might not capture all poor neurodevelopmental outcomes. Rusli et al. used the Trivedi MRI scoring system and found no significant association between MRI injury score and outcomes of cerebral palsy or death by two years of age (p=0.350) [19]. Trivedi et al. 2017 developed a scoring system that sums MRI injury severity across the brainstem, cortex, white matter, and five subcortical locations as follows: the globus pallidus, putamen, caudate nucleus, thalamus, and PLIC to generate a composite MRI injury score [18]. Trivedi et al. assessed their metric for association with outcomes using the BSID-III across 57 neonates with HIE. To evaluate their scoring metric, they dichotomized outcomes by labeling neonates with BSID-III scores <85 as poor outcomes and BSID-III scores >85 as good outcomes. Trivedi et al. found that their MRI injury score was significantly associated with poor cognitive outcomes (p<0.001) and motor outcomes (p<0.012). In contrast, Rusli et al. only evaluated the scoring system using a cohort of 19 neonates and their functional outcome metric was the development of cerebral palsy by two years of age. These discrepancies may explain the poor performance of the Trivedi scoring system in the study by Rusli et al. [19].

Another study evaluated MRI injury patterns independently of a specific MRI scoring system to prognosticate HIE outcomes [37]. They found that the development of post-neonatal epilepsy was associated with subcortical injuries involving the basal ganglia, thalamus, and brainstem. Lakatos et al. considered both MRI and magnetic resonance spectroscopy (MRS) findings as potential predictors of outcomes [37]. For MRI, they considered three patterns of injury as follows: basal ganglia-thalamus, watershed pattern, and total brain injury as well as the presence of concomitant intracerebral hemorrhage (ICH). For MRS, they considered a high lactate/N-acetyl aspartate (Lac/NAA) ratio on MRS as indicative of injury. The outcome was assessed using a BSID-II score at 18-26 months. On multivariate regression, they found that infants with these MRI or MRS patterns had higher odds of poor outcome (odds ratio: 6.23; CI 95%: 1.26, 30.69; p=0.025) than those who were HIE negative on both MRS and MRI. Interestingly, a concomitant intracerebral hemorrhage was not significantly associated with worse outcomes.

Quantitative scoring for lesion burden: Quantitative MRI measures were also assessed as predictors of outcome. In a study of 107 term neonates using diffusion-weighted MR images, Chang et al. determined the lesion size and the number of lesions in the NICHD injury score locations [28]. They found that DWI-MRI lesion sizes >500 pixels and lesion counts between 14 and 40 were both independently associated with poor outcomes. Chintalapati et al. in 2021 and Takenouchi et al. in 2010 assessed diffusion restriction using the apparent diffusion coefficient (ADC) [29,30]. Chintalapati et al. assessed the ADC in the striatum and thalamus and found that an average striatal ADC less than 1.014 × 10-3 mm^2^/s across free-drawn regions of interest in the left and right striatum had 100% specificity and 70% sensitivity for the development of dystonic cerebral palsy. In addition, an average thalamic ADC of less than 0.973 × 10-3 mm^2^/s across free-drawn regions of interest in both the left and right thalamus had 100% specificity and 80% sensitivity for the development of dystonic cerebral palsy. Finally, using a cohort of 34 neonates, Takenouchi et al. in 2010 compared infants who had restricted diffusion changes in the splenium of the corpus callosum to those without changes. They found that those with restricted diffusion in the splenium had higher rates of poor neurocognitive outcomes (p=0.002). Restricted diffusion in the splenium had a positive predictive value of 90% for poor motor outcomes and a negative predictive value of 71% [30].

Discussion

This systematic review was conducted to identify features for predicting neurodevelopmental outcomes in term and near-term neonates with HIE who received therapeutic hypothermia. This review focuses on articles that apply EEG and MRI since they are two of the most frequently implemented modalities for assessing neonatal brain structure and function in clinical practice. Functional outcomes were assessed in the reviewed studies primarily using standardized metrics such as the BSID at 18-24 months or by evaluating for the presence of conditions such as cerebral palsy or post-neonatal epilepsy. Multiple EEG and MRI features were predictive of neurodevelopmental outcomes.

EEG Patterns Associated With Poor Functional Outcome

Four EEG features observed during therapeutic hypothermia were associated with poor functional outcomes as follows: an abnormal EEG background pattern, interhemispheric asynchrony, lack of sleep-wake cycle recovery, and increased seizure burden. In the period after therapeutic hypothermia, burst and interburst characteristics were useful predictors. Because these EEG features manifest during and shortly after the therapeutic hypothermia window, they may be biomarkers of secondary energy failure.

The Effect of Cooling on MRI Pseudonormalization

MRI patterns were a bit more complex to assess, in part because cooling may cause discrepancies due to pseudonormalization, particularly in scoring systems that evaluate MRI diffusivity [40,41]. Pseudonormalization occurs when the MRI diffusivity returns to baseline after dipping below baseline due to acute injury [40]. Thus, injuries would no longer appear on MRI diffusion sequences. In non-cooled neonates, MRI pseudonormalization occurs around 6-8 days post-injury, whereas cooling pushes pseudonormalization out to 10-11 days [40,41]. Additionally, MRI injury severity scores in the basal ganglia and watershed region were found to be significantly lower in cooled neonates than in non-cooled neonates [42].

Metabolism and MRI Injury

To constrain potential heterogeneity in results, the articles selected in this review only included HIE cohorts that received therapeutic hypothermia. These articles showed that injury within deep subcortical structures, particularly the basal ganglia and thalamus, were consistently associated with poor outcomes. This pattern may exist because the basal ganglia and thalamus are among the most metabolically active brain regions in term neonates [43]. This finding is corroborated by regional hyperperfusion in the basal ganglia and thalamus, which was captured using MRI-arterial spin labeling [43]. Thus, these structures seem particularly susceptible to changes in brain perfusion, with the extent of their damage correlating with the severity of hypoxic injury.

Relationship of EEG Patterns to MRI Activity

There is also the question of how EEG compares to MRI for outcome prediction, since EEG may be more accessible than MRI in certain settings, for example, in infants not stable enough to be transported for an MRI or in hospital units without ready access to an MRI scanner [44]. Severe EEG background abnormalities, attenuated EEG power, and electrographic seizure burden all correlate with MRI injury severity [21,45,46]. Despite these correlations, it is unclear if EEG patterns can be used to predict specific MRI injury patterns. Clarifying these questions will allow clinicians to ascertain the extent to which EEG can serve as a biomarker for both MRI injury location and neurodevelopmental outcome.

Clinical Implications of Better Prognostic Algorithms

The ultimate goal of identifying the most accurate and useful prognostic features is to provide clinicians and families with sound data on which to base care decisions and to aid clinicians in counseling families about their newborn’s probable neurodevelopmental outcome [47]. Families must make critical decisions in the acute neonatal period that influence the continuation of life-sustaining therapies and overall goals of care for the neonate. These decisions often include whether to place tracheostomy and gastrostomy tubes, whether to escalate to invasive ventilation, and whether to initiate extracorporeal membrane oxygenation (ECMO). These decisions depend, in part, on the etiology, severity, and prognosticated outcome of hypoxic-ischemic injury. Higher fidelity prognostication provided by sensitive and specific biomarkers may allow clinicians to feel more confident in the counseling they provide to families and allow families to feel more confident in their decisions in the acute setting.

Limitations of the Included Studies

Sample size:* *This review also identified limitations that may serve as opportunities to better characterize prognosticators for neonatal HIE in future studies. Many included studies have small sample sizes; for example, seven of the 20 articles reviewed had fewer than 30 subjects (appendix 2-4). This limits the number of variables that can be assessed in a particular case series and raises the risk that a few outliers can bias the results. Larger studies, or even a reanalysis of several previous datasets with a predefined protocol, may help confirm which markers are the best, independent predictors of outcome.

MRI interpretation: While MRI has proven important for prognostication, its use must be standardized in clinical practice to ensure consistent interpretation. For example, MRI pseudonormalization after the first week of life can cause injuries to appear less severe [31]. Basti et al. in their study (appendix 4), assessed MRIs at various time windows (range: 5-30 days). MRIs performed after one week of life may be susceptible to the effects of diffusion pseudonormalization, which can underestimate injury severity. This approach may have caused MRI results to vary [26]. MRI-based prognostic features should therefore be defined for a specific imaging time window for clinical practice. In addition, most MRI scoring systems focus on scoring the extent of cortical injury as opposed to assessing particular areas of the cortex that may have differential effects on prognosis [18,21,48]. There is a need for validated scoring systems that allow prognostication of specific types of disability based on injury to particular cortical areas.

Long-term follow-up metrics: One more subtle limitation of the studies reviewed is the focus on long-term outcome data at 18-24 months. While this follow-up window likely gives an accurate description of the ultimate disability a child will face, it does not capture the full demographic spectrum of those at risk. Neonates with less access to clinical care may be more likely to have HIE and are more susceptible to follow-up loss [49]. These neonates may also have less access to early intervention therapies that can influence functional outcomes. Swearingen et al. in 2020 showed a follow-up loss of 62% across a cohort of 237 neonatal patients with rates varying significantly across different demographic strata such as median income and race [49]. High rates of follow-up loss are concerning because assessments like the Bayley Scales compare infant outcomes using normative scores that are established using cohorts of infants that can receive follow-up.

Towards a Focus on Injury Location for Prognostication

The current guidelines for outcome prognostication using MRI recommend assessing injury immediately after cooling and within 10 days of life [50]. The guidelines rely on the NICHD and Barkovich systems that score injury as an aggregate across the basal ganglia and thalamus, internal capsule, white matter, and watershed zones [48,51]. More detailed scoring systems, such as the Rutherford et al. and Weeke et al. systems, take into account specific injury locations such as the corpus callosum and particular areas of the cortex but have not been incorporated into prognostication guidelines [52,53]. When all four scoring systems were compared, the more detailed scoring systems produced stronger correlations to adverse neurodevelopmental outcomes [54]. These findings suggest that knowing specific injury locations might produce better outcome predictions and expanding the range of locations evaluated may yield new insights for outcome prognostication in neonatal HIE [30]. Since long-term follow-up at 18 months and above is considered standard of care and most articles included were retrospective (n=16), they largely did not address follow-up, potentially biasing results. One exception was the prospective study by Sewell et al. [34]. They identified that infants retained had worse pathology (i.e. lower Appearance, Pulse, Grimace, Activity, and Respiration {APGAR} scores). Thus, they speculated that their follow-up assessments were focused on infants in greatest need of care.

The limitations of the present study include relying on data from a limited number of databases to identify potentially eligible studies, as well as interrater reliability in the subjective analysis of MRI and EEG studies.

## Conclusions

This systematic review amalgamates the clinical features taken from both EEG and MRI that are associated with long-term neurodevelopmental outcomes in neonates undergoing therapeutic hypothermia for HIE. We have identified specific injury patterns in these neuromonitoring tools that correlate with specific markers for developmental prognosis. Currently, 72-hour therapeutic hypothermia coupled with continuous EEG monitoring and followed by MRI within seven days of hypoxic injury is the standard of care for full-term infants with HIE. High-fidelity prognostic EEG and MRI features can help guide the clinical management of HIE and escalation of care. Ultimately, the various features identified will need to be combined into a multimodal model for outcome prediction that can be easily used by clinicians to benefit patients and their families at the NICU bedside.

## Appendices

Appendix 1

**Table 2:** Preferred Reporting Items for Systematic Reviews and Meta-Analysis. From the PRISMA 2020 statement: an updated guideline for reporting systematic reviews (http://www.prisma-statement.org/) [12].

Section and topic	Item#	Checklist item	The location where item is reported
Title			
Title	1	Identify the report as a systematic review	Page 1
Abstract			
Abstract	2	See the PRISMA 2020 for abstracts checklist	Doc included
Introduction			
Rationale	3	Describe the rationale for the review in the context of existing knowledge	Page 1
Objectives	4	Provide an explicit statement of the objective(s) or question(s) the review addresses	Page 1
Methods			
Eligibility criteria	5	Specify the inclusion and exclusion criteria for the review and how studies were grouped for the syntheses	Page 2
Information sources	6	Specify all databases, registers, websites, organizations, reference lists, and other sources searched or consulted to identify studies. Specify the date when each source was last searched or consulted	Page 2
Search strategy	7	Present the full search strategies for all databases, registers, and websites, including any filters and limits used	Page 2 and supplementary table, appendix 1
Selection process	8	Specify the methods used to decide whether a study met the inclusion criteria of the review, including how many reviewers screened each record and each report retrieved, whether they worked independently, and if applicable, details of automation tools used in the process	Page 2
Data collection process	9	Specify the methods used to collect data from reports, including how many reviewers collected data from each report, whether they worked independently, any processes for obtaining or confirming data from study investigators, and if applicable, details of automation tools used in the process	Page 2
Data items	10a	List and define all outcomes for which data were sought. Specify whether all results that were compatible with each outcome domain in each study were sought (e.g., for all measures, time points, analyses), and if not, the methods used to decide which results to collect	Page 2
	10b	List and define all other variables for which data were sought (e.g., participant and intervention characteristics, funding sources). Describe any assumptions made about any missing or unclear information	Page 2
Study risk of bias assessment	11	Specify the methods used to assess risk of bias in the included studies, including details of the tool(s) used, how many reviewers assessed each study and whether they worked independently, and if applicable, details of automation tools used in the process	Page 2 and second, third, and fourth supplementary tables
Effect measures	12	Specify for each outcome the effect measure(s) (e.g., risk ratio, mean difference) used in the synthesis or presentation of results	Page 2
Synthesis methods	13a	Describe the processes used to decide which studies were eligible for each synthesis (e.g., tabulating the study intervention characteristics and comparing against the planned groups for each synthesis {item #5})	Page 2
	13b	Describe any methods required to prepare the data for presentation or synthesis, such as handling missing summary statistics, or data conversions	Page 2 and first figure
	13c	Describe any methods used to tabulate or visually display results of individual studies and syntheses	Page 2
	13d	Describe any methods used to synthesize results and provide a rationale for the choice(s). If meta-analysis was performed, describe the model(s), method(s) to identify the presence and extent of statistical heterogeneity, and software package(s) used	Page 2
	13e	Describe any methods used to explore possible causes of heterogeneity among study results (e.g., subgroup analysis, meta-regression)	N/A
	13f	Describe any sensitivity analyses conducted to assess robustness of the synthesized results	N/A
Reporting bias assessment	14	Describe any methods used to assess risk of bias due to missing results in a synthesis (arising from reporting biases)	Page 2
Certainty assessment	15	Describe any methods used to assess certainty (or confidence) in the body of evidence for an outcome	N/A
Results			
Study selection	16a	Describe the results of the search and selection process, from the number of records identified in the search to the number of studies included in the review, ideally using a flow diagram	Page 3 and first figure
	16b	Cite studies that might appear to meet the inclusion criteria, but which were excluded, and explain why they were excluded	First figure
Study characteristics	17	Cite each included study and present its characteristics	Pages 4-7, second, third, and fourth supplementary tables
Risk of bias in studies	18	Present assessments of risk of bias for each included study	Pages 4-7
Results of individual studies	19	For all outcomes, present, for each study: (a) summary statistics for each group (where appropriate) and (b) an effect estimate and its precision (e.g., confidence/credible interval), ideally using structured tables or plots	Second, third, and fourth supplementary tables
Results of syntheses	20a	For each synthesis, briefly summarize the characteristics and risk of bias among contributing studies	Second, third, and fourth supplementary tables
	20b	Present results of all statistical syntheses conducted. If meta-analysis was done, present for each the summary estimate and its precision (e.g., confidence/credible interval) and measures of statistical heterogeneity. If comparing groups, describe the direction of the effect	Pages 6-10
	20c	Present results of all investigations of possible causes of heterogeneity among study results	Page 6-10, 13
	20d	Present results of all sensitivity analyses conducted to assess the robustness of the synthesized results	Pages 6-10
Reporting biases	21	Present assessments of risk of bias due to missing results (arising from reporting biases) for each synthesis assessed	Page 6-10
Certainty of evidence	22	Present assessments of certainty (or confidence) in the body of evidence for each outcome assessed	Pages 6-10
Discussion			
Discussion	23a	Provide a general interpretation of the results in the context of other evidence	Pages 11-15
	23b	Discuss any limitations of the evidence included in the review	Pages 11-15
	23c	Discuss any limitations of the review processes used	Pages 11-15
	23d	Discuss implications of the results for practice, policy, and future research	Pages 11-15
Other information			
Registration and protocol	24a	Provide registration information for the review, including register name and registration number, or state that the review was not registered	N/A
	24b	Indicate where the review protocol can be accessed, or state that a protocol was not prepared	N/A
	24c	Describe and explain any amendments to information provided at registration or in the protocol	N/A
Support	25	Describe sources of financial or non-financial support for the review, and the role of the funders or sponsors in the review	Page 16
Competing interests	26	Declare any competing interests of review authors	Page 16
Availability of data, code and other materials	27	Report which of the following are publicly available and where they can be found: template data collection forms; data extracted from included studies; data used for all analyses; analytic code; any other materials used in the review	Supplementary table, Page 6, appendix 1 (database search string)

Appendix 2

**Table 3:** Preferred Reporting Items for Systematic Reviews and Meta-Analysis abstract. From the PRISMA 2020 statement: an updated guideline for reporting systematic reviews (http://www.prisma-statement.org/) [12].

Section and topic	Item#	Checklist item	Reported (Yes/No)
Title			
Title	1	Identify the report as a systematic review	Yes
Background			
Objectives	2	Provide an explicit statement of the main objective(s) or question(s) the review addresses	Yes
Methods			
Eligibility criteria	3	Specify the inclusion and exclusion criteria for the review	Yes
Information sources	4	Specify the information sources (e.g., databases, registers) used to identify studies and the date when each was last searched	Yes
Risk of bias	5	Specify the methods used to assess risk of bias in the included studies	Yes
Synthesis of results	6	Specify the methods used to present and synthesize results	Yes
Results			
Included studies	7	Give the total number of included studies and participants and summarize relevant characteristics of studies	Yes
Synthesis of results	8	Present results for main outcomes, preferably indicating the number of included studies and participants for each. If meta-analysis was done, report the summary estimate and confidence/credible interval. If comparing groups, indicate the direction of the effect (i.e., which group is favored)	Yes
Discussion			
Limitations of evidence	9	Provide a brief summary of the limitations of the evidence included in the review (e.g., study risk of bias, inconsistency, and imprecision)	Yes
Interpretation	10	Provide a general interpretation of the results and important implications	Yes
Other			
Funding	11	Specify the primary source of funding for the review	No
Registration	12	Provide the registered name and registration number	No

Appendix 3

**Table 4:** Reproducible search string used for PubMed database to find papers focusing on monitoring and prognostic modalities for neonates with HIE. HIE: hypoxic-ischemic encephalopathy

PubMed database search strings
((((hypoxic ischemic encephalopathy) AND (neonate)) AND (monitor)) AND (brain))) AND (therapeutic hypothermia OR hypothermia)
("hypoxic ischaemic encephalopathy"[All Fields] OR "hypoxia ischemia, brain"[MeSH Terms] OR ("hypoxia ischemia"[All Fields] AND "brain"[All Fields]) OR "brain hypoxia-ischemia"[All Fields] OR ("hypoxic"[All Fields] AND "ischemic"[All Fields] AND "encephalopathy"[All Fields]) OR "hypoxic ischemic encephalopathy"[All Fields]) AND ("infant, newborn"[MeSH Terms] OR ("infant"[All Fields] AND "newborn"[All Fields]) OR
"newborn infant"[All Fields] OR "neonatal"[All Fields] OR "neonate"[All Fields] OR "neonates"[All Fields] OR "neonatality"[All Fields] OR "neonatals"[All Fields] OR "neonate s"[All Fields]) AND ("monitor s"[All Fields] OR
"monitorable"[All Fields] OR "monitored"[All Fields] OR "monitoring"[All Fields] OR "monitoring s"[All Fields] OR "monitoring, physiologic"[MeSH Terms] OR ("monitoring"[All Fields] AND "physiologic"[All Fields]) OR "physiologic monitoring"[All Fields] OR "monitor"[All Fields] OR "monitorings"[All Fields] OR "monitorization"[All Fields] OR "monitorize"[All Fields] OR "monitorized"[All Fields] OR "monitors"[All Fields]) AND ("brain"[MeSH Terms] OR
"brain"[All Fields] OR "brains"[All Fields] OR "brain s"[All Fields]) AND ("cooled"[All Fields] OR
"therapeutic hypothermia"[All Fields] OR "therapeutic hypothermias"[All Fields] OR "cools"[All Fields] OR
("hypothermia"[MeSH Terms] OR "hypothermia"[All Fields] OR "hypothermias"[All Fields] OR "hypothermia s"[All Fields]))

Appendix 4

**Table 5:** Summary of articles assessing EEG features (n=8). BG/T: basal ganglia/thalamus; aEEG: amplitude-integrated EEG; TH: therapeutic hypothermia therapy; CNV: continuous normal voltage; DNV: discontinuous normal voltage; BS: burst suppression; LV: low voltage; FT: flat trace; ADC: apparent diffusion coefficient; MDI: mental developmental index; IBI: interburst interval; GMFCS: Gross Motor Function Classification system; CP: cerebral palsy; HOL: hour of life; TSB: total seizure burden; MSB: maximum seizure burden; MRS: magnetic resonance spectroscopy; AEDs: anti-epileptic drugs; GFMDS: Griffiths Mental Development Scales; PICU: pediatric intensive care unit; T1W: T1-weighted; T2W: T2-weighted; TTDC: time to discontinuous; TTN: time to normalization; TTC: time to cycling; RCT: randomized control trial; WML: white matter lesion; NICHD: National Institute of Child Health and Human Development

Studies	Setting (country)	Patients included	Outcome criteria	Features assessed	Important modality features associated with outcome (assessment metric)	Comments
Csekő et al. (2013) [33]	NICU Semmelweis University (Hungary)	70 term neonates GA (>37 weeks)	Abnormal outcome=BSID-II (MDI<70) or death at 18-24 months	aEEG background SWC on aEEG	Abnormal aEEG background had PPV of 0.92 for predicting an abnormal outcome at 60 hours, while the PPV for an abnormal outcome with no SWC was 0.73	Retrospective study, independent and blinded reviewers
Dereymaeker et al. (2019) [23]	NICU of UZ Leuven (Belgium)	19 neonates (GA 36-41 weeks)	Abnormal outcome=death, CP, or BSID-II (<70) at 24 months	Dynamic IBI scored for severity from 1 to 4	Dynamic IBI severity score from 18-24 hours of life (AUC=0.93)	Retrospective study
Fitzgerald et al. (2018) [24]	NICU of Children’s Hospital of Philadelphia (United States)	93 neonates (mean GA 38.7 weeks)	Abnormal outcome=language and motor delays at 24 months	Epileptic seizure exposure in EEG background	High epileptic seizure exposure predicts abnormal language development (p=0.04), and moderate/severely abnormal EEG background predicts motor delay (p=0.01)	Retrospective study, also showed seizure exposure could predict abnormal MRIs (p=0.02)
Kharoshankaya et al. (2016) [11]	Cork University Maternity Hospital (Ireland)	47 neonates (median GA=40.7 weeks)	Abnormal outcome measured via BSID-III, GMDS, and CP at 24-28 months	EEG seizure burden (TSB and MSB)	TSB>40 minutes and MSB>13 minutes (p=0.001 and p=0.003, respectively)	Retrospective study
Koskela et al. (2021) [22]	University College London Hospitals (United Kingdom)	41 neonates (GA 36.3-41.6 weeks)	Abnormal outcome scored using BSID-III at median of 24 months (range 12-36 months)	EEG cortical burst power (8-30 Hz) recovery at post-natal day 3 and above	High burst power associated with worsened outcomes across channels C3, C4, T4, (p<0.05) independent of MRI	Retrospective study, controlled for MRI injury severity to show independence
Leroy-Terquem et al. (2017) [36]	Necker-Enfants Malades Hospital (France)	40 neonates (GA≥36 weeks)	Abnormal outcome = WHO disability score at 24 months	EEG asynchrony within the first 7 days of life	EEG asynchrony had 97% sensitivity and 80% specificity for predicting major disability	Retrospective study, choice of 1.5 seconds as threshold for burst separation could be further justified
Sewell et al. (2018) [34]	Children’s National Health Systems (United States)	80 neonates (GA≥35 weeks)	Abnormal outcome scored via BSID-II and BSID-III at 18 months	aEEG background, aEEG latency factors TTDC, TTN, TTC	aEEG background pattern was significant (p<0.005), and aEEG latency factors predicted outcomes with a sensitivity of 0.944 and a specificity of 0.852	Prospective study also looked at short-term outcome metrics using MRI severity as a metric
Takenouchi et al. (2011) [25]	New York-Presbyterian Hospital (United States)	29 neonates (GA≥36 weeks)	Outcome-based on BSID-II MDI and ambulation without or support at ≥18 months	Acquisition of SWC during the first 144 hours of life	Failure to acquire SWC by 120 hours in severe HIE neonates is associated with poor outcomes (p=0.02)	Retrospective study, also used ROC to predict that 120 hours is most sensitive for outcome prediction but with a low AUC of 0.53

Appendix 5

**Table 6:** Summary of articles assessing MRI features (n=8); all studies were retrospective. GA: gestational age; BSID: Bayley Scales of Infant Development; GMFCS: Gross Motor Function Classification System; CP: cerebral palsy; ADC: apparent diffusion coefficient; NICHD: National Institute of Child Health and Human Development; MRS: magnetic resonance spectroscopy; ICH: intracerebral hemorrhage; Lac/NAA: high lactate/N-acetyl aspartate; HIE: hypoxic-ischemic encephalopathy; GFMDS: Griffiths Mental Development Scales; PPV: positive predictive value; NPV: negative predictive value; MDI: mental developmental index

Studies	Setting (country)	Patients included in study	Outcome criteria	Modalities assessed	Features assessed	Important modality features associated with outcome (assessment metric)	Comments
Chang et al. (2020) [28]	Seoul St. Mary’s Hospital (Korea)	107 neonates (GA≥35 weeks)	Abnormal outcome = BSID-III scores <85, or death at 18-24 months	MRI (DWI) within 10 days of life	Lesions scored via NICHD scoring system, lesion size (pixels)	MRI lesion size <100 and >500 (p<0.05), MRI lesion counts <2 and between 14 and 40 (p<0.05), NICHD stages 0 to 2A (p<0.05)	Retrospective study
Chintalapati et al. (2021) [29]	St. Louis Children’s Hospital (United States)	50 neonates (GA≥35 weeks)	Abnormal outcome = dystonia or spasticity at up to 5 years	MRI (DWI) at day 4-5 of life	Striatal and thalamic ADC	Striatal ADC <1.014 × 10^−3^ mm^2^/s (100% specificity and 70% sensitivity), thalamic ADC <0.973 × 10-3 mm^2^/s (100% specificity and 80% sensitivity)	Retrospective study, somewhat restrictive region of interest by only focusing on striatal and thalamic regions
Hayakawa et al. (2018) [31]	Red Cross Kyoto Daiichi Hospital (Japan)	17 neonates (mean GA = 38.3 weeks)	Abnormal outcome = major disability via GMFCS for CP & standard neurologic exam At 18 months	MRI (DWI) scored twice at mean day 3 of life and mean of day 10 of life	DWI pseudonormalization in MRI from week 2 of life	Week 2 pseudonormalization negativity (100% sensitivity, 100% specificity)	Retrospective study, small sample size
Lakatos et al. (2019) [37]	Semmelweis University (Hungary)	108 (GA≥36 Weeks)	Abnormal outcome = BSID-II score	MRI (DWI), MRS within 7 days of life	MRI injury pattern ICH on MRI, Lac/NAA ratio	Presence of HIE via both MRI and MRS was significantly associated with poor outcomes (p=0.0246)	Retrospective study, concomitant ICH had no significant effect on outcomes
Mastrangelo et al. (2019) [38]	Sapienza University of Rome (Italy)	29 neonates (GA≥34 weeks)	Abnormal outcome = GFMDS-III global quotient <85 at both 12 and 24 months	MRI (DWI) mean = 5.7 days (range 1-20 days)	MRI scored via Bednarek severity scores	PPV of MRI global score at 12 and 24 months=36.36% and 50%, respectively, NPV of MRI at 12 months=93.75%	Retrospective study, included single neonate with GA below 34 weeks
Trivedi et al. (2017) [18]	St. Louis Children’s Hospital (United States)	57 neonates (GA≥35 weeks)	Bayley-III score at 18-24 months	MRI (T1W, T2W, DWI) scored twice at mean of 4 days and mean of 10 days. Worse score used	Injury severity score across subcortical structures, white matter, cortex, cerebellum, and brainstem	Increased MRI injury grade was significantly associated with poor cognitive and motor outcomes (p<0.001, p<0.012)	Prospective study, most patients' scores (32 of 41) did not change between early and late scans. Later scans generally had worse grades
Rusli et al. (2019) [19]	NICU of Universiti Kebangsaan Malaysia Medical Center (India)	19 neonates (GA≥36 weeks)	Abnormal outcome = death or CP based on clinician notes at 24 months	MRI (T1W, T2W, DWI) within 2 weeks of life	MRI scored using Trivedi et al. 2017 scoring system	Trivedi scoring system was not significantly associated with poor outcome (p=0.350)	Retrospective study, used different outcome metrics to Trivedi et al. (Trivedi et al. used BSID-III)
Takenouchi et al. (2010) [30]	NICU at New York-Presbyterian Hospital (United States)	34 neonates (GA≥36 Weeks)	Abnormal outcome BSID-III MDI score at 18 months	MRI (DWI) within 7 days of life	ADC of splenium, corpus callosum	Restricted diffusion in splenium is significantly associated with poor outcomes (p=0.002)	Retrospective study

Appendix 6

**Table 7:** Summary of articles assessing both EEG and MRI features (n=4). EEG: electroencephalogram; aEEG: amplitude-integrated EEG; CNV: continuous normal voltage; DNV: discontinuous normal voltage; BS: burst suppression; LV: low voltage; FT: flat trace; PLIC: posterior limb of the internal capsule; BG/T: basal ganglia/thalamus; WML: white matter lesion; BSID: Bayley Scales of Infant Development; AEDs: anti-epileptic drugs; NICHD: National Institute of Child Health and Human Development; HIE: hypoxic-ischemic encephalopathy; cEEG: continuous electroencephalography; CP: cerebral palsy; TSB: total seizure burden; HOL: hour of life

Studies	Setting (country)	Patients included in study	Outcome criteria	Modalities assessed	Features assessed	Important modality features associated with outcome (assessment metric)	Comments
Basti et al. (2020) [26]	NICU San Salvatore Hospital in L’Aquila (Italy)	30 term neonates	Abnormal outcome = death, spastic quadriplegia and BSID-III at 18-24 months	aEEG MRI (T1, T2, DWI), MRI taken within 4 weeks of life	Seizure burden aEEG background (CNV, DNV, BS, LV, FT), MRI injury pattern (PLIC, multifocal, BG/T, WML)	Significant features (p<0.05) = high seizure burden, abnormal aEEG over 48 hours, and abnormal MRI pattern	Prospective study, MRI taken within 4 weeks of life (median=17 days), used aEEG alone to identify seizures
Lin et al. (2021) [27]	Seoul St. Mary's Hospital (South Korea)	97 neonates (GA≥35 weeks)	Abnormal outcome=BSID-II score	MRI within 10 days of life, aEEG	Clinical seizures (evidenced by the use of AEDs), aEEG background NICHD MRI pattern	Abnormal aEEG associated with poor outcome (p<0.05) BG/T and PLIC lesion groups in 2A and 2b associated with abnormal outcomes (p<0.001)	Retrospective study, MRI taken (≤10 days of life), also showed seizure severity was associated with BG/T lesions
Peeples et al. (2021) [35]	Children’s Hospital and Medical Center (United States)	486 neonates (mean GA=38.8 weeks)	Abnormal outcome based on BSID-III and GMFCS score at a median of 23.8 months (interquartile range 18.6-27.9 months)	EEG, aEEG, MRI (DWI) within first 7 days of life	MRI HIE severity (either cortical or deep gray injury), EEG background, aEEG background	Combo of either severe grade HIE or abnormal aEEG/cEEG at 24 hours is associated with poor outcomes (p<0.001)	Retrospective study
Weeke et al. (2016) [21]	Wilhelmina Children's Hospital (Netherlands)	26 neonates (GA mean 40.4 weeks)	Abnormal outcome via BSID-III, CP, epilepsy, hearing or vision loss, death at a median of 26 months (range 16-32 months)	EEG, MRI (T1, T2, DWI) within the first two weeks of life	EEG, TSB, and EEG, MRI pattern via Barkovich score	EEG background at 36 HOL, TSB, and MRI all associated with outcomes (p=0.009, p=0.036, p<0.001, respectively)	Multicenter randomized trial, no multivariate analysis
